# The Mechanical Reinforcing Mechanism and Self-Healing Properties of Biomimetic Hybrid Cement Composites via In-Situ Polymerization

**DOI:** 10.3390/ma18163763

**Published:** 2025-08-11

**Authors:** Wenhui Bao, Jian Zhao, Bumin Guo, Shuan Li, Jinwei Shen, Mengyuan Liu, Jingmin Han, Susu Xing, Miaomiao Hu, Jintang Guo

**Affiliations:** 1 China Oilfield Services Limited, Tianjin 300450, China; baowh@cnooc.com.cn (W.B.); zhaojian12@cnooc.com.cn (J.Z.); guobm@cnooc.com.cn (B.G.); lishuan@cnooc.com.cn (S.L.); shenjw5@cnooc.com.cn (J.S.); 2 School of Chemical Engineering and Technology, Tianjin University, Tianjin 300350, China; brunhilds7@163.com (M.L.); jmhan2021@tju.edu.cn (J.H.); xingss@tju.edu.cn (S.X.); 3 Zhejiang Institute of Tianjin University, Shaoxing 312300, China; 4 Haihe Laboratory of Sustainable Chemical Transformations, Tianjin 300192, China

**Keywords:** cementitious materials, self-healing cement, in situ polymerization, biomimetic composite

## Abstract

Addressing the inherent brittleness of cement to mitigate infrastructure failures stemming from cracking is imperative. To accomplish both early crack resistance and subsequent self-healing capabilities, a biomimetic microstructure composed of a sodium polyacrylate (CSPA) network interwoven with hydration products was developed. The calcium-enriched polymer network formed via in situ polymerization of sodium acrylate (ANa) can enhance the mechanical properties of cement and achieve efficient self-healing of cracks. The porous structure of sodium polyacrylate (PANa) formed in pore solution at room temperature to simulate cement hydration conditions was observed by scanning electron microscopy (SEM). Feature peaks found by Fourier transform infrared (FTIR) spectroscopy as well as confocal Raman microscopy (CRM) suggested that ANa was polymerized successfully. Notably, CSPA samples demonstrated a remarkable 104% increase in flexural strength, attributed to the efficient transmission and dissipation of external forces along the polymer network embedded within the cement matrix. Additionally, after a 28-day hydration, CSPA specimens exhibited enhanced compressive strength compared to blank cement samples. This enhancement stems from the formation of a uniform polymer network, which effectively decreased the porosity and densified the microstructure of cement. Moreover, this organic–inorganic hybrid structure contributes to efficient crack healing, as the calcium-rich polymer network binds calcium ions and promotes the generation of healing products. The healing products consist of calcium hydroxide (CH), CaCO_3_ (aragonite), C-S-H (calcium–silicate–hydrate), and PANa.

## 1. Introduction

Due to their low costs, high compressive strength, good flame retardance, and easy availability, cement-based materials are widely used in various construction works, such as bridge construction, housing construction, road building, and so on. However, their inherent low tensile strength and high brittleness elevate the risk of cracking and compromise the durability of infrastructure [1]. To guarantee safety during application and extend the lifespan of infrastructure, numerous strategies have been implemented to reinforce cement and repair the cracks. Researchers have endeavored to incorporate flexible and elastic polymers [2] into cement matrices to mitigate the occurrence of unforeseen cracking. These polymers, including rubber [3,4], epoxy resin [5,6,7], and polyacrylamide [8,9], are utilized in the form of latex and powder for cement modification. Robisson et al. [10] mixed hydrogenated nitrile butadiene rubber with cements to make a cement–rubber composite with the advantages of ductility and stiffness combined. Fonseca et al. [11] selected jute fiber and micro/nanofiber as reinforcements for cement-based composites, aiming to improve mechanical performance. While these reported methods can indeed mitigate cracking to a certain extent, they often lead to a decrease in the compressive strength of cement, primarily owing to the inherent strength disparities between the toughening materials and the cement itself.

To solve the structural failure caused by cracking, various self-healing agents have been reported [12]. Zheng et al. [13] investigated the impact of encapsulation-based spores on cement self-healing and found that the long-term resistance decreased to a certain level. Ying et al. [14] effectively healed cracks in cement using a crack response capsule, albeit at the cost of reduced compressive strength. Zheng et al. [15] prepared autolytic mineral microspheres that modulated hydration progression and enhanced the self-healing property of cement-based composites, albeit with a decrease in strength. These materials can heal cracks under different conditions, and the achievement of self-healing and the increasing healing efficiency are often accompanied by a decline in the mechanical properties of cement. Consequently, there is a pressing need for a method that can simultaneously enhance both mechanical properties and healing efficiency.

Nacre, a natural biological material comprising 95% inorganic CaCO_3_ and 5% polymer, exhibits outstanding strength and toughness [16]. This remarkable advantage is attributed to the thin layers of polymer that effectively bond the regularly stacked CaCO_3_ [17]. To enhance the mechanical properties of cement, this structure has been replicated via in situ polymerization. Through an innovative biomimetic layered structure design, Chen et al. [18] successfully developed a multi-layered cement–hydrogel composite material with breakthrough performance. By integrating the dual dynamic bonding mechanisms of Fe^3+^–carboxyl coordination bonds and hydrogen bond networks, it realizes the synergistic optimization of high toughness, low thermal conductivity, and excellent self-healing performance. Liu et al. [19] prepared a biomimetic anisotropic hydrogel, namely a alginate/polyacrylamide/halloysite nanotube hybrid hydrogel (SA/AM/HNTs-RDC), as a self-healing agent. This hydrogel can react with multiple ions in the pore solution, stimulating the massive production of healing products and promoting their dense deposition. The aim is to enhance the self-healing capability of cement-based infrastructure and extend its service life. Yin et al. [20] developed a plug-in network structure via in situ polymerization of zirconium phosphate–acrylamide, leading to a great increase in flexural strength. These polymer additives and in situ polymerization can only control the growth of cracks at the prophase, while the healing of the structure after cracking has not been reported. Sodium acrylate (ANa) is a highly reactive monomer [21] that enables in situ polymerization under the relatively harsh conditions of cement hydration. Chen et al. [22] demonstrated that the carboxyl groups in ANa form salt bridges within the cement matrix, thereby strengthening the interfacial bonding and improving the flexural strength of the cement–polymer composite. Resan et al. [23] explored the feasibility of sodium polyacrylate as a cement additive, and the results revealed that adding sodium polyacrylate has a positive impact on the compressive strength and consistency of cement–sand mortar. Chen et al. [24] studied the rheological properties of cement paste modified by in situ polymerization with different dosages of sodium acrylate. To sum up, the ssodium polyacrylate (PANa) network formed by ANa can enhance the flexural strength of cement [25]. Additionally, the PANa network can seal cracks through swelling induced by water absorption and promote the formation of calcium carbonate. Han et al. [26] achieved efficient healing of existing cracks through the synergistic reaction between cement matrix activation and organic polymerization of sodium acrylate (ANa) by using sodium silicate, sodium silicate/sodium acrylate, and sodium silicate/sodium polyacrylate as healing agents. Given these advantages, PANa has the potential to significantly improve both the mechanical properties and healing efficiency of cement composites. The data comparison of the aforementioned relevant literature is shown in Table 1.

Inspired by nature, this study introduces sodium acrylate into the cement paste system and effectively initiates its in situ polymerization reaction in the cement matrix. A PANa network is formed in cement to construct biomimetic hybrid cement composites. The copolymerization process of ANa during cement hydration at room temperature was verified via Fourier transform infrared (FTIR) spectroscopy and confocal Raman microscopy (CRM). Subsequently, the three-dimensional polymer network was observed by scanning electron microscopy (SEM). Various properties, including flexural strength, hydration heat, and rheological properties, were measured to elucidate the impact of the polymers on the cement. To delve into the effect of PANa on calcium ion enrichment, the elemental composition of the healed cement was determined using energy dispersive spectrometry (EDS). To estimate the repair efficiency, a 3D digital microscope was used for a visualization test. A comprehensive investigation of the microstructure and contents of the healing products was conducted using SEM, FTIR, thermogravimetric analysis (TG), and X-ray diffraction (XRD).

## 2. Material and Methods

### 2.1. Material

The cement used in this study is G-class oil well cement, and Table 2 shows the contents of the cement, which were determined with an X-ray fluorescence spectrometer (XRF, S4 Pioneer, Billerica, MA, USA). Sodium acrylate (ANa, AR), sulfite (Na_2_SO_3_, AR), and ammonium persulfate (APS, AR) were used in the polymerization.

### 2.2. Preparation Progress

#### 2.2.1. Preparation of PANa

Before implementation, the appropriate polymerization conditions of ANa were observed. It is demonstrated that polymerization can occur at room temperature by using ANa as monomer (M), APS as initiator (I) and Na_2_SO_3_ as co-initiator (I’). The ratio of I and M was set as 1.8%, and the ratio of I’ and M was 1.5%. First, 2.0 g Ana was added into a beaker of 20 mL of deionized water (DI water) on a stirrer. This was stirred constantly until the monomer dissolved, and then APS was added into the ANa solution to obtain the mixture. Subsequently, the mixed solution was reacted for 3 h under stirring with Na_2_SO_3_ solution dipped. The reaction solution was dialyzed in 2000 mL of DI water to remove the impurities and purify the polymer. The purified solution transformed into solid PANa polymer via lyophilization in a vacuum freeze-dryer. To simulate the reaction in cement pastes, a pore solution was used to replace DI water in another experiment.

**Figure 1:**
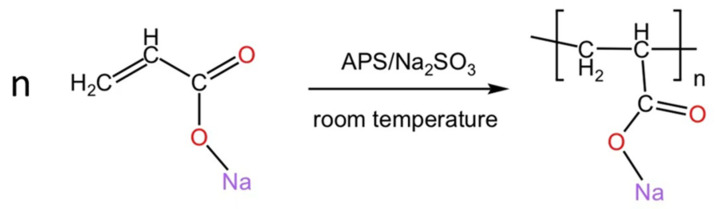
Equation for the polymerization reaction of ANa.

The equation for the polymerization reaction of ANa is shown in Figure 1.

#### 2.2.2. Preparation of CSPA

The composite cement material CSPA was prepared via the addition of polymerization to the cement. The G-class high-sulfur-resistant oil well cement was used to make all the paste, of which w/c was set as 0.44. M, I, and I′ were added to water. Then, the solution was used to make the paste. Chinese standard (GBT 1346–2024) [27] is the execution standard of cement paste mixing operations. The composites obtained were named for CSPA-1, CSPA-2, CSPA-3, and CSPA-4 according to the different component ratios in the cements. Table 3 lists the mix design of all CSPA samples.

Table 4 lists all CSPA samples.

### 2.3. Test and Characterization

#### 2.3.1. Structural Characterization of PANa

To confirm the occurrence of polymerization at room temperature, the obtained product was characterized via FTIR (IS 50, FEI Company, Hillsboro, OR, USA) and CRM (Labram HR Evolution, HORIBA Jobin Yvon, Paris, France). Changes in surface functional groups of polymerization products were characterized by FTIR in the range of 400–4000 cm^−1^, using 32 scans with a resolution of 4 cm^−1^.

CRM was used to calculate the ratio of the change in C-C single bonds to C-C double bonds in the system before, during, and after reaction. This was carried out in the range of 50–2000 cm^−1^ with a 532 nm laser.

#### 2.3.2. Effects of ANa Polymerization on Properties of CSPA

For the determination of the flexural strength of the CSPA samples after 3 days of curing, the fresh mixed slurry was solidified in a mold with a size of 40 mm × 40 mm × 100 mm at 30 °C and hydrated for 3 days. Then, the cubes were loaded with a compression testing machine to determine their flexural strength and choose the optimum dosage. An integrated machine for compressive and flexural strength (YAM-200D, Jinan Nai’er Testing Machine Co., Ltd., Jinan, China) was employed to conduct the flexural strength test, and the maximum load at the moment when the cement stone was broken was recorded. The calculation formula is as per Equation (1):

(1) $$P_{f}=\frac{1.5FL}{b^{3}}$$

where $P_{f}$ represents the flexural strength of the cement stone, *F* is the maximum load before the cement stone breaks, *L* denotes the distance between the two fulcrums, and *b* is the side length of the cross-section of the sample block.

Specimens with a size of 50.8 mm × 50.8 mm × 50.8 mm were cast to measure the compressive strength of the CSPA after curing.

According to GBT 19139-2012 [28], rheological properties tests of blank cement sample paste and CSPA were conducted using a Hack torque rheometer. After being fully stirred, the rotor speed rose from 3 r/s to 300 r/s, then decreased from 300 r/s to 3 r/s. The entire deceleration process is divided into 13 steps, with each step held for 10 s to ensure a stable shear stress. As the data for the shear stress at different rotor speeds were obtained, the power-law function was used to fit the data points to obtain the function as per Equation (2):

(2) $$\tau =k{\frac{du}{dv}}^{n}$$

where *τ* is the shear stress, *k* is the consistency index, *n* is the liquidity index and $\frac{du}{dv}$ is the shear rate.

To investigate the effect of in situ polymerization on the cement hydration process, an automatic cement hydration calorimeter (Yite 12959-08, Wuhan Yite Instrument Co., Ltd., Wuhan, China) was used to monitor the hydration exothermic rate and total heat release of both the pure cement paste and cement paste with polymerization introduced. The water bath was kept at the test temperature throughout. The obtained heat flow curves, including the data recordings, can reveal the effect of polymerization on the hydration of cement. Prior to the start of the test, cement particles were first thoroughly mixed with mixing water in accordance with the paste mixing procedure. For the pure cement paste system, tap water was used as the mixing water. For the cement paste system involving polymerization, the mixing water was pre-dissolved with ANa monomers and the initiator APS-Na_2_SO_3_. After the paste mixing was completed, the resulting slurry was quickly poured into the insulated inner container of the tester. The container was sealed with a wooden stopper and paraffin, then placed in a constant-temperature water tank (bath temperature: 20 °C) and fixed. Finally, after inserting the thermocouple, the test program was initiated to monitor the temperature change in the cement paste during hydration.

To explore the impact of the polymerization reaction on the pore structure of cement, pore structure tests were conducted on cement samples cured for 28 days. The core of the crushed cement after the mechanical performance test was ground and then sieved by a 200-mesh sieve. The powder sample was dried at 90 °C for 4 h. Afterwards, the pore structure was tested via a Tristar physical adsorption analyzer. The hydration products were characterized via SEM and TG(TG-209).

Preparation and testing of SEM samples: Conductive adhesive was evenly applied to the sample stage, and a small amount of sample was placed on the conductive adhesive. Samples that were not firmly adhered were gently blown off. The testing voltage was set at 3 kV.

#### 2.3.3. Self-Healing Properties of CSPA

To observe the progress of crack healing more intuitively, specimens with a size of 18 mm × 16 mm × 18 mm were cast for the recovery of self-healing efficiency of blank cement and CSPA-3 after 28 days of curing. Cracks with a width of 50 µm were created artificially via a scalpel. Then, the samples were cured in pore solution at 30 °C for 3 d, 7 d, 14 d, and 28 d. Calcium leaching due to water can induce microcracks in cement as well as degradation of physical properties [29]. Ions leach from cement into water, which means that the water is transformed into a solution containing ions [30]. The leaching process is too long to actualize. Thus, a pore solution was chosen as the curing environment to simulate the work conditions in which cement is used in water for a long time, such as dams. After being cured for different numbers of days, the samples were observed via VHX-2000. The width of the cracks was measured via software called Nano Measurer. Healing efficiency was calculated based on the measured crack width.

To obtain the self-healing products in the cracks and further investigate the self-healing mechanism, the collection process of the healing product samples is described as follows. After being cured for 28 days, cement samples were split in half and then pieced together to the original state. Broken cement samples were cured in pore solution for another 28 days. After that, healing products for tests were collected from the cement fracture surface.

The composition of the healing products was discerned through FTIR, XRD, and TG. The FTIR test was performed using 32 scans with a resolution of 4 cm^−1^ in a transmission model ranging from 400 to 4000 cm^−1^. The TG test was carried out in a nitrogen atmosphere with a N_2_ flow rate of 30 mL/min. It ranged in test temperature from 30 to 1000 °C, and the heating rate was 10 °C/min. XRD testing was conducted within the range of 5 to 70°/2θ, with a step size of 0.01° and a speed of 10°/min.

## 3. Results and Discussion

### 3.1. Structure of PANa

#### 3.1.1. FTIR Analysis

**Figure 2:**
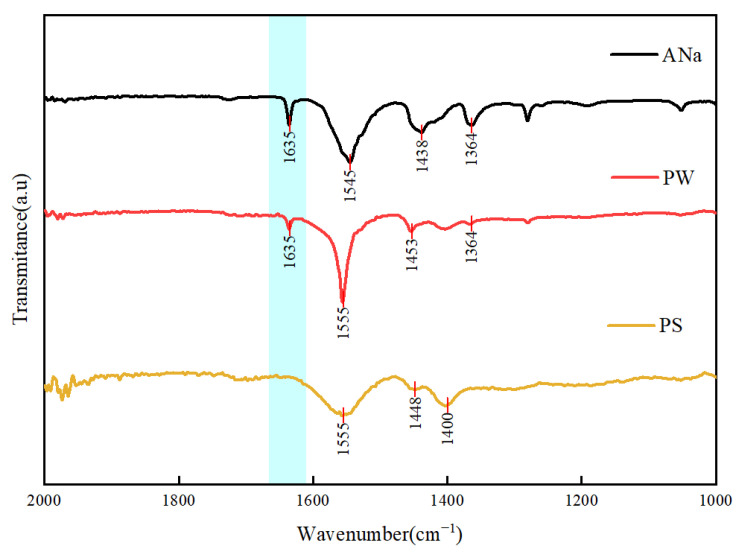
FITR of ANa, PANa composed in water (PW), and PANa composed in pore solution (PS).

To confirm the polymerization of PANa during the hydration process, a combination of FTIR, CRM, and SEM was utilized. FTIR spectra curves of ANa and PANa composed in water (PW) and pore solution (PS) were drawn, as shown in Figure 2. In the FTIR analysis of ANa, stretching peaks were detected at around 1545 cm^−1^ and 1364 cm^−1^, indicative of the asymmetric and symmetric vibrations of COO^−^ groups, respectively. Furthermore, ANa exhibited absorption bands near 1438 cm^−1^, which are ascribed to the symmetrical stretching vibration of COO^−^ [31]. The peak at 1635 cm^−1^ corresponded to the asymmetric stretching vibrations of C=C bonds within ANa [32]. The FITR spectrum of PW exhibited asymmetric and symmetric stretching peaks at approximately 1555 cm^−1^ and 1364 cm^−1^, respectively, along with a COO^−^ symmetrical stretching vibration at 1453 cm^−1^. Compared to ANa, the intensity of the peak at 1635 cm^−1^ decreased, suggesting that a significant portion of the C=C bonds participated in the polymerization reaction. As for PS, peaks at 1555 cm^−1^, 1448 cm^−1^, and 1400 cm^−1^ corresponded to the asymmetric vibrations, symmetric vibrations, and symmetrical stretching vibrations of COO^−^ groups, respectively. Notably, the disappearance of the characteristic peak at 1635 cm^−1^ indicated that the C=C bonds in Ana were converted into C-C single bonds through the polymerization of monomers, confirming the successful formation of PANa in the pore solution.

#### 3.1.2. Raman Analysis

**Figure 3:**
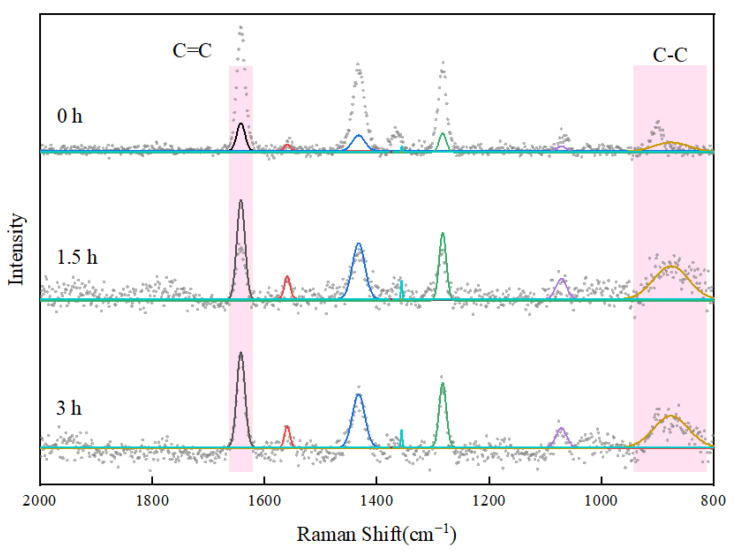
Raman spectra (RS) of ANa in the pore solution at 0 h, 1.5 h, and 3 h of the copolymerization reaction.

To confirm the copolymerization of ANa in the pore solution, the ratio of C=C bonds to C-C single bonds within the reaction system was quantified via Raman spectra. Figure 3 illustrates the test results of the samples collected after different reaction times. In the three curves, all the peaks are observed at a similar Raman shift, but the differences lie in the intensity of each peak. The peaks located at 1432 cm^−1^ correspond to the stretching of COO^−^ groups. The peaks at about 1360 cm^−1^ and 1067 cm^−1^ are attributed to the bending vibration of (CH_2_) and wagging vibration of (CH_2_), respectively. Additionally, peaks at about 1280 cm^−1^ are observed, which can be assigned to the bending vibration of C-O-H groups. Notably, the feature peak at 1640 cm^−1^ corresponds to the stretching of C=C bonds [33], while the peak observed at 870 cm^−1^ is indicative of the stretching vibration of C-C single bonds [34].

To visually illustrate the changes in peak intensity, the peak areas were quantified through fitting calculations and are presented in Table 5. The data reveals that the percentage of the peak area at approximately 1640 cm^−1^ generally presents a decreasing trend, with a reduction from 31.67% to 23.13%. Conversely, the percentage of the peak area at about 870 cm^−1^ increased, growing from 7.44% to 32.88%. Consequently, the ratio of the peak area at about 1640 cm^−1^ to the peak area at about 870 cm^−1^ decreased from 4.26 to 0.70, which is less than one-sixth of its original numerical value. This phenomenon suggests that the proportion of C=C bonds in the system decreased and the proportion of C-C single bonds increased during the process of polymerization. In other words, the C=C bonds of ANa transferred into C-C single bonds of PANa over time. The combined results from FTIR and RS confirm the polymerization of PANa.

#### 3.1.3. Zeta

**Figure 4:**
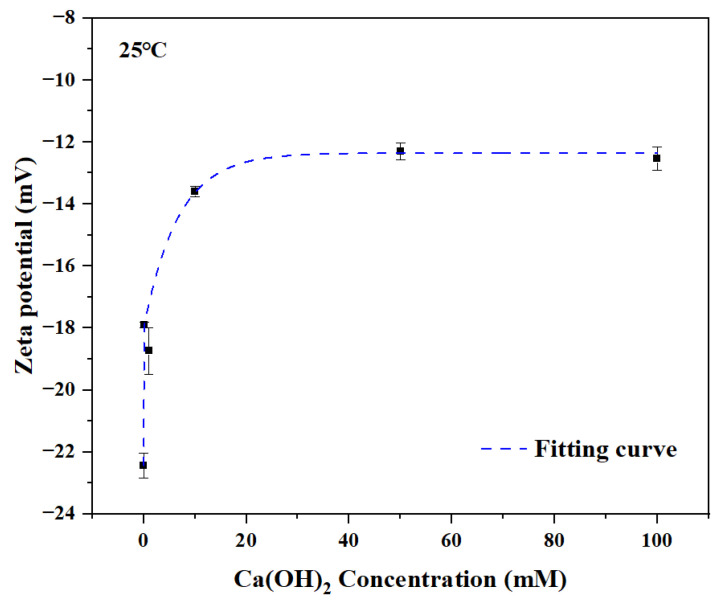
Variations in zeta potential of sodium polyacrylate solutions with different additions of calcium hydroxide.

To eliminate the interference of other elements and components in the cement on the experimental results, we selected calcium hydroxide (Ca(OH)_2_), the main hydration product of cement, to replace the cement matrix for the experiment. The complexation between PANa and Ca^2+^ in cement was explored by testing the changes in zeta potential of sodium polyacrylate solutions with different contents of calcium hydroxide added. The results are shown in Figure 4. With the increase in calcium ion concentration, the zeta potential increases. This is because the carboxyl groups (-COO^−^) on the molecular chain of sodium polyacrylate make it negatively charged in aqueous solution, so the zeta potential of colloidal particles (or molecular aggregates) in the pure PANa system is usually negative. As divalent cations, calcium ions (Ca^2+^) will have an electrostatic attraction with -COO^−^ on the PANa chain and even form coordination bonds, which leads to the neutralization of negative charges on the surface of PANa, thus causing a significant change in zeta potential.

#### 3.1.4. SEM Analysis

**Figure 5:**
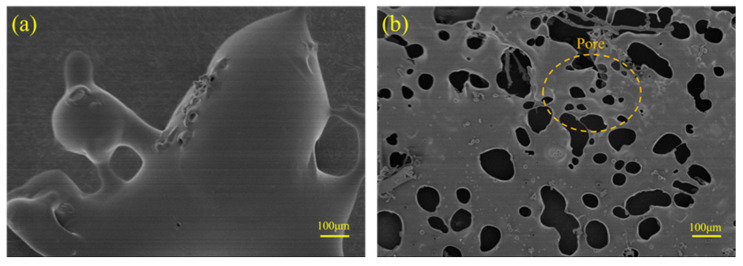
SEM images of PANa in water (**a**) and in the pore solution (**b**).

To confirm the formation of the PANa network in the pore solution, the structure of the polymer was measured via SEM. Figure 5 illustrates the microcosmic structure of PW and PS. PW was in a clumpy shape with a smooth surface, while PS was in a porous network structure. The existence of calcium in a certain concentration range reduces the size and range of effective interaction between two polymer layers [35]. This phenomenon arises due to the formation of calcium bridges in the pore solution, resulting from the interaction between calcium ions and PANa. These bridges subsequently decreased the effective charge of the polymer layers, thereby promoting the network configuration of PS rather than the block-like structure observed in PW. The polymer network is constructed through the complexation of calcium ions with two carboxylic acid groups. Prior studies have already documented such interactions [36]. Notably, PS boasts a significantly larger surface area compared to PW, enabling it to offer a plethora of binding sites for calcium ions. The structural divergence between PW and PS underscores the capacity of PANa to sequester calcium ions, thereby facilitating efficient calcium enrichment in cementitious materials. This observation of the polymer–calcium complexation network structure substantiates the binding affinity of PANa towards calcium ions. Similar methodologies have been employed to demonstrate the calcium enrichment efficacy of polymeric network structures [37,38].

### 3.2. Effects of ANa Polymerization on the Hydration Process of CSPA

#### 3.2.1. Mechanical Properties of Cement Composites

**Figure 6:**
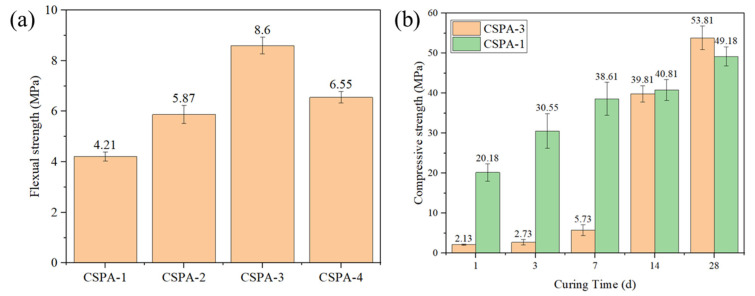
(**a**) The flexural strength of CSPA after curing for 3 days; (**b**) compressive strength of CSPA-3 and CSPA-1 cured for different days.

Figure 6a represents the flexural strengths of the cement with different amounts of ANa. Toughness is defined as the ability of a material to absorb energy during plastic deformation until the fracture [39]. In situ polymerization has a positive effect on the great improvement in cement flexural strength. On one hand, the concurrent occurrence of polymerization and hydration reactions facilitated the formation of an organic–inorganic interpenetrating network structure within the cement matrix [25]. As the external force transmits and dissipates along the polymer network, the resistance and tenacity of cement are improved [22]. On the other hand, during the formation of hydrogen bonds among carboxyl groups in PANa, protonated silicate, and H_2_O molecules, Ca^2+^ and Na+ ions serve as bridging agents between oxygen atoms in C-S-H (calcium–silicate–hydrate) and those in silicate structures. These hydrogen bonds, along with O-Ca-O and O-Na-O linkages, strengthen the bonding interface between the polymer and cement, preventing interface cracking. The result indicates a trend in which the flexural strength initially increases and then decreases with an increase in the dosage of Ana. When the dosage is below the optimum level, the incompatibility between inorganic cement and organic polymer leads to weakened toughness and a weaker interface interaction of the incorporated polymers, thereby reducing the overall mechanical performance. When the dosage exceeds the optimum level, the adverse influence of the polymer on the mechanical strength of the cement becomes more pronounced than the beneficial dissipation effect of the interpenetrating networks on external forces [22]. The optimum dosage of ANa is 4 wt%, and all subsequent experiments were conducted using this dosage.

Figure 6b illustrates the compressive strength of the CSPA-3 samples after curing for various days. The results demonstrate a temporal improvement in the resistance of cement. Specifically, when the CSPA-3 specimens were cured for less than 14 days, their compressive strength was lower than that of the blank cement sample due to deferred hydration. Upon reaching a curing period of 14 days, the compressive strength of the sample approached that of blank cement. After 28 days of curing, the compressive strength of the CPSA-3 cement matrix was slightly higher than that of the blank cement. This improvement in the resistance of hardened cement pastes with Ana was because of the graded hydration. A higher water-to-cement (w/c) ratio facilitated more complete cement hydration. The delayed hydration increased the w/b (water-to-binder) ratio of the remnant cement, thereby promoting the overall degree of hydration [40].

#### 3.2.2. Rheological Properties of CSPA

**Figure 7:**
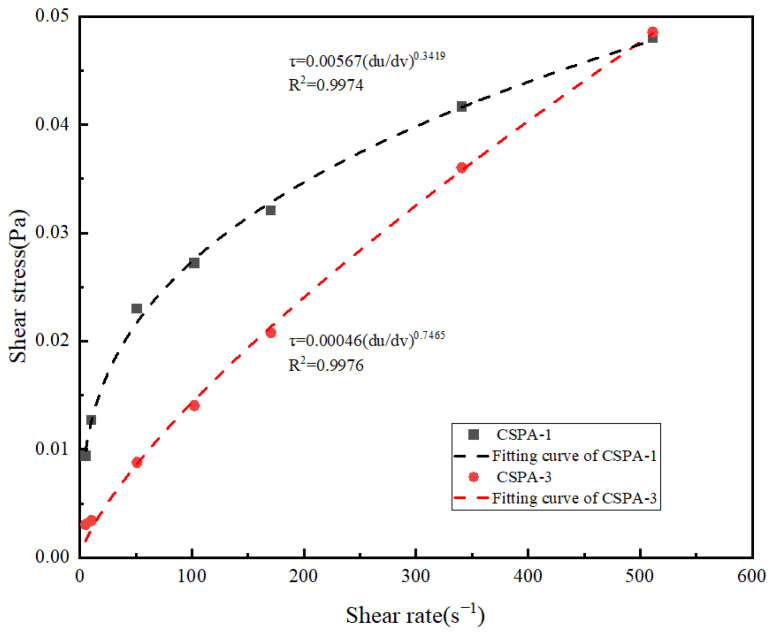
Rheological properties of CSPA-1 and CSPA-3.

**Figure 8:**
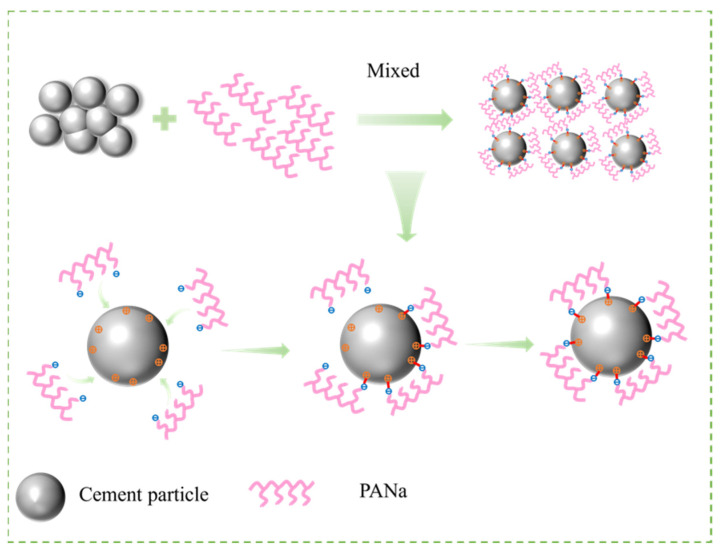
Mechanism of the enhancement of cement dispersing performance.

The rheological characteristics of the cement slurry are portrayed in Figure 7. It clearly presents that the liquidity index lies within a range greater than 0 but less than 1, indicating that both cement pastes are categorized as pseudoplastic fluids. The consistency index of CSPA-3 (0.00046) is much lower compared to that of the blank cement sample (0.00567), suggesting that the viscosity of the blank cement slurry surpasses that of CSPA-3. This indicates that the incorporation of PANa, formulated from ANa, serves to reduce the viscosity of the cement slurry. The reduction results from the carboxyl within the polymer chain of PANa, which binds to calcium ions via chelation on the cement surface [41]. This binding enhances electrostatic repulsion and steric hindrance, thereby improving the dispersion of cement particles and enhancing the rheological properties of the cement. The mechanism is shown in Figure 8.

#### 3.2.3. Hydration Progress

**Figure 9:**
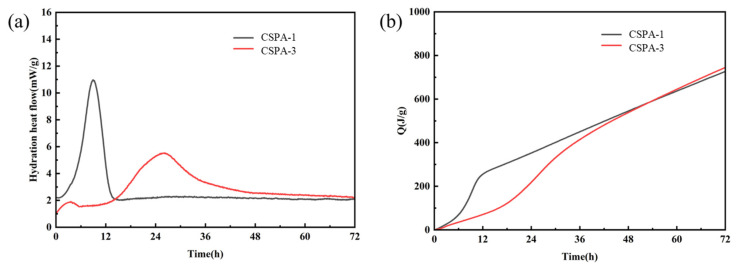
(**a**) Hydration heat flow curve of pure cement and cement with polymerization reaction; (**b**) hydration heat in total of pure cement and cement with polymerization reaction.

The thermal evolution of direct hydration between cement and water is shown in Figure 9. The hydration of Portland cement can be divided into five stages: pre-induction, induction, acceleration, deceleration, and steady-state [42]. The induction period was not observed in the exothermic curve of the pure cement paste (Figure 9a) because this stage commences immediately upon cement–water contact and has a relatively short duration. In contrast, the introduction of polymerization retarded the hydration process, leading to the observation of the first exothermic peak. The incorporation of ANa notably postponed the exothermic peak of the hydration heat flow, and the peak appears roughly at 26 h, later than the peak associated with CSPA-1. The polymerization process of ANa resulted in a reduction in both the crest values of heat flow during cement hydration, as depicted in Figure 9a [43]. An abundance of ANa further contributed to the formation of multilayer polymer films that were interposed between cement particles and hydration products, thereby retarding the cement hydration process. Additionally, PANa absorbed free water in the system, validly inhibiting the reaction between cement and water at its source, which further delayed cement hydration [25]. Figure 9b shows that in the early stage, the cumulative heat release of the cement paste with sodium acrylate polymerized in situ is significantly lower than that of the pure cement paste. This further indicates that the introduction of polymerization retards the hydration of cement. These findings suggest that polymerization acts as an inhibitor of cement hydration at early stages, facilitating the establishment of a graded hydration mechanism. Specifically, the in situ polymerization of ANa in the early stage delays hydration, reduces microcracks, ensures structural integrity, and reserves reaction space for subsequent hydration. In the later stage, the cement continues to undergo hydration reactions, and its hydration products fill the pores, realizing the transformation of the pore structure and enhancing the long-term strength. Coupled with the strengthening effect of the sodium polyacrylate–calcium complex network, a “synergistic enhancement” is formed together with the continuously generated hydration products, ensuring the sustained growth of cement strength and its long-term stability. This is consistent with the results in Figure 6.

#### 3.2.4. Analysis of Hydration Products of Cement

**Figure 10:**
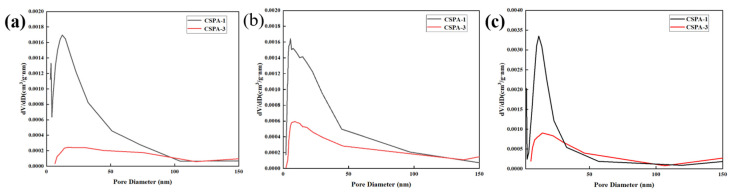
Pore structure of samples: (**a**) cements cured for 3 d; (**b**) cements cured for 14 d; (**c**) cements cured for 28 d.

Figure 10 illustrates the pore structure of the cement stone. The intensity of the peaks in the curve is positively correlated with the pore volume. Table 6 shows the pore data of CSPA-1 and CSPA-3. During the first 14 days, the quantity of pores with a diameter smaller than 100 nm in CSPA-3 is significantly smaller than in the blank cement. After 28 days of curing, CSPA-3 still exhibits a higher density compared to the blank cement sample. This indicates that the PANa network successfully filled the pores in the cement, reducing the mesopore content and thereby improving the microstructure. This enhancement in microstructure increases CSPA-3’s ability to absorb energy during plastic deformation, leading to higher flexural strength.

**Figure 11:**
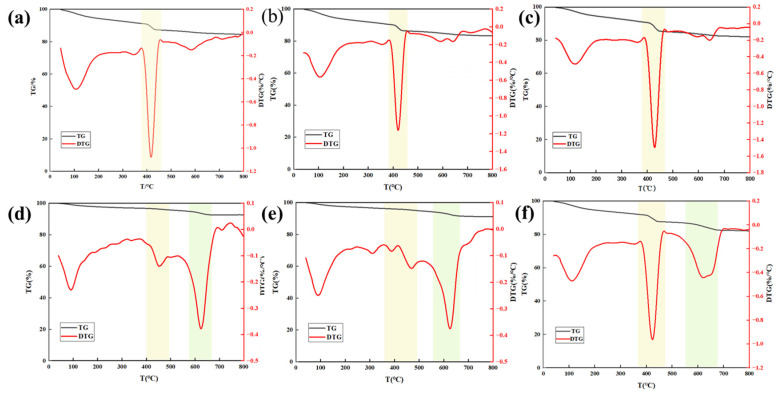
TG/DTG curve of hydration products: (**a**) blank cement cured for 3 d; (**b**) blank cement cured for 14 d; (**c**) CSPA-1 cured for 28 d; (**d**) CSPA-3 cured for 3 d; (**e**) CSPA-3 cured for 14 d; (**f**) CSPA-3 cured for 28 d.

TG was conducted to analyze the composition of the hydration products. The weight loss between 100–200 °C is attributed to the loss of bound water in the C-S-H gels. Figure 11a–c show a significant mass reduction within the temperature range of 400–440 °C, corresponding to the decomposition of C-H in the blank cement. Figure 11d–f display the TG results for CSPA-3. In contrast to the blank cement, CSPA-3 shows notable mass loss in the range of 550–650, which corresponds to the decomposition of CaCO_3_. The mass loss percentages of CSPA-1 and CSPA-3 in different weight loss stages are shown in Table 7. The comparative analysis revealed that the weight loss peak intensities of CSPA-3 between 100–200 °C and 400–440 °C were lower than those of CSPA-1, whereas the peak intensity between 550–650 °C was higher. This can be attributed to the fact that the carboxyl groups (-COO^−^) in PANa molecules can adsorb onto the surface of cement particles, forming a polymer film that impedes the contact between water molecules and clinker minerals, thereby delaying early-stage hydration. The delayed cement hydration reduces the formation of early-age C-S-H. However, with prolonged hydration, the improved dispersibility of cement particles enables more clinker minerals to participate in the reaction, potentially increasing the late-stage C-S-H content. Secondly, PANa forms stable complexes with Ca^2+^ in the cement paste, reducing the concentration of free Ca^2+^ in the liquid phase and slowing down the precipitation rate of calcium hydroxide (CH). Consequently, the CH content is low at early ages, resulting in low decomposition weight loss. As hydration proceeds, the content of CH increases in the later stage (28 days). However, due to the continuous inhibition of hydration by PANa, the weight loss peak intensity of CSPA-3 is still lower than that of CSPA-1. Compared with CSPA-1, CSPA-3 has higher porosity due to insufficient hydration, which allows CO_2_ to diffuse easily, accelerates carbonation, and leads to more calcite (CaCO_3_) formation. Hence, the weight loss of CSPA-3 increases in this stage.

**Figure 12:**
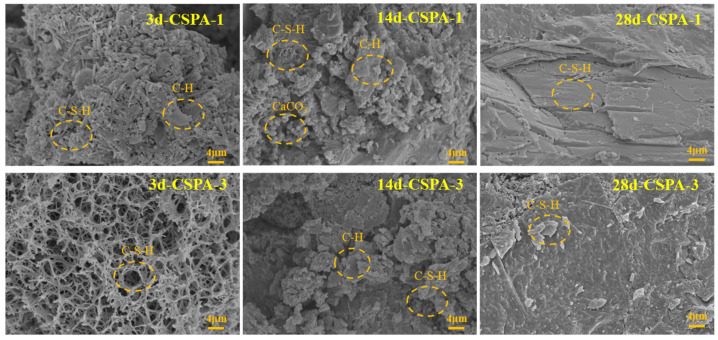
SEM images of cement hydration products after different curing days.

To verify the effects of PANa on cement hydration, hydration products specifically from the cracking regions of the samples were collected for detailed examination. Figure 12 presents the SEM images of these hydration products at various curing durations. After 3-day curing, the hydration products of the blank cement consisted of acicular (needle-like) C-S-H (calcium–silicate–hydrate), whereas those of the cement containing PANa exhibited porous networks [44], further confirming the occurrence of polymerization within the cement matrix. Upon curing for 28 days, the 28d-Neat system revealed the presence of layer-packed CH. The existence of such a layer-packed structure renders the cement susceptible to interlayer slipping under external forces. In contrast, the 28d-PANa system exhibited a denser structure in comparison with the blank cement sample, elevating both the resistance and tenacity of the cement. This observation aligns with the findings presented in Figure 10. The evenly distributed polymer network formed in the cement via in situ polymerization concurrently bolstered both the resistance and toughness of the cementitious material.

### 3.3. Self-Healing Properties of CSPA

#### 3.3.1. Apparent Self-Healing Performance of CSPA

**Figure 13:**
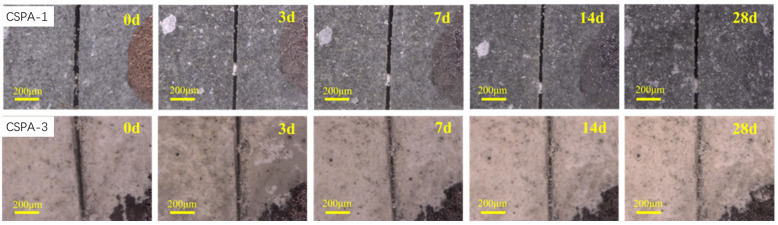
Crack healing images of different cement samples.

Figure 13 depicts pictures of the cracked samples cured for different days shot via digital microscopy. Here, “BC” denotes the blank cement sample, while “C-3” represents CSPA-3. In the blank cement specimen, the white healing products originated from within the crack and filled only a minor portion of it. Notably, after 28 days of curing, the crack remained incompletely sealed. This outcome suggests that a 28-day curing period is inadequate for the healing of microcracks in the blank cement. In contrast to the blank cement, the crack in the CSPA-3 specimen exhibited a greater propensity for healing after just 7 days of curing and achieved complete healing after 28 days of curing.

**Figure 14:**
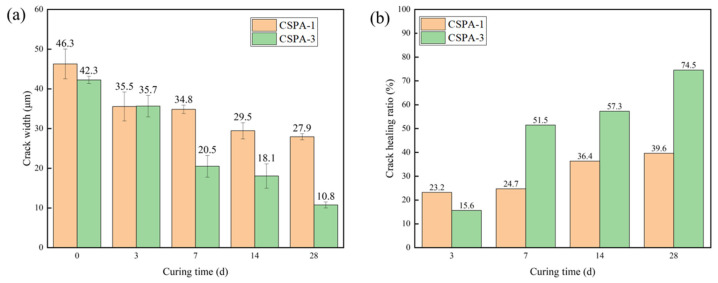
(**a**) The crack width after different curing days; (**b**) the crack healing ratio after different curing days.

Figure 14 shows the quantified healing efficiency of the cement samples. Figure 14a presents the average width of cracks in the cement after curing for different durations. For the blank cement samples, the crack width decreased from 46.3 μm to 29.5 μm during the initial 14 days, a reduction of 16.8 μm. In the following 14 days, the crack width decreased by only 1.6 μm. In contrast, for CSPA-3, the crack width decreased from 42.3 μm to 10.5 μm during the first 14 days, a reduction of 24.2 μm. Over the next 14 days, the crack width continued to decrease by 7.3 μm. Figure 14b shows the crack healing ratio of the cements after curing for different periods. The crack healing ratio of CSPA-3 reached 74.5% after 28 days, while the ratio for blank cement was only 39.6%. These results clearly demonstrate that CSPA-3 exhibits persistent healing ability and higher healing efficiency compared to blank cement.

**Figure 15:**
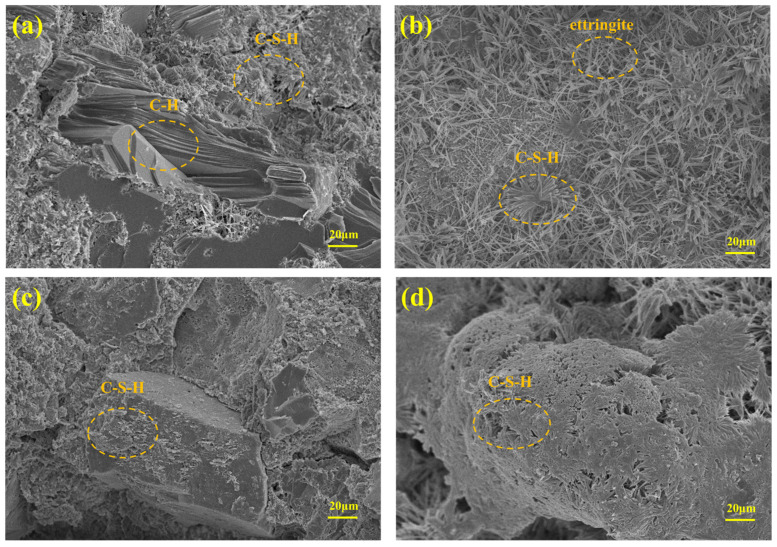
SEM images of cement fragments: (**a**) CSPA-1 before healing; (**b**) CSPA-1 after healing; (**c**) CSPA-3 before healing; (**d**) CSPA-3 after healing.

To explore the healing mechanism, the healing products were further analyzed via SEM. As shown in Figure 15, the repair products in blank cement sample primarily consisted of stick-like C-S-H, forming a relatively loose structure. In contrast, the repair products on the surface of cement containing PANa were primarily interconnected in a slice-like fashion. The PANa served as a template for the growth of the healing products, leading to a dense packing of the healing products. This is because the carboxyl functional group of PANa provide numerous active sites for interaction with calcium ions. This interaction also facilitated the provision of nucleation sites for the healing products [37].

#### 3.3.2. Apparent Self-Healing Performance of CSPA

**Figure 16:**
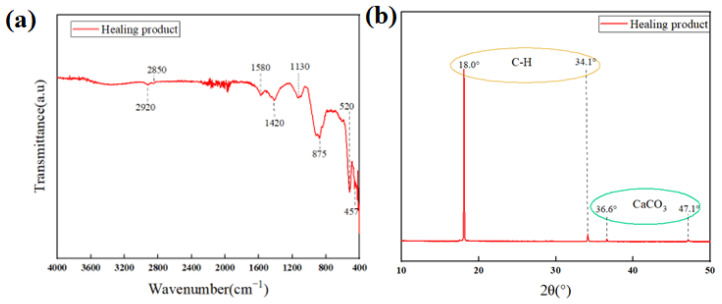
(**a**) FTIR of CSPA-3 healing product; (**b**) XRD of CSPA-3 healing product.

FTIR, XRD, and TG analyses were performed to characterize the contents and structures of the healing products formed in CSPA-3. Figure 16a shows the FTIR curve of the healing products. The feature peaks observed at 2920 cm^−1^ and 2850 cm^−1^ correspond to the asymmetric and symmetric vibrations of (CH_2_), respectively, which indicates the existence of PANa in the healing products. The bands at about 875 cm^−1^ and 1420 cm^−1^ belong to out-of-plane bending and the asymmetric stretching of CO_3_^2−^, respectively, providing evidence for the formation of calcium carbonate. The peak at about 1130 cm^−1^ is due to SO_2_^4−^ in ettringite [45]. The symmetric vibration of Ca-O could be observed at 520 cm^−1^ [46]. The bands at 457 cm^−1^ were linked to Si-O bond bending vibrations in C-S-H. Figure 16b exhibits peaks at 18.0° [47] and 34.1°, corresponding to the presence of C-H. The peaks at 36.6° and 47.1° correspond to aragonite [48]. In conclusion, the components of the healing products contain C-H, C-S-H, CaCO_3_, and ettringite.

**Figure 17:**
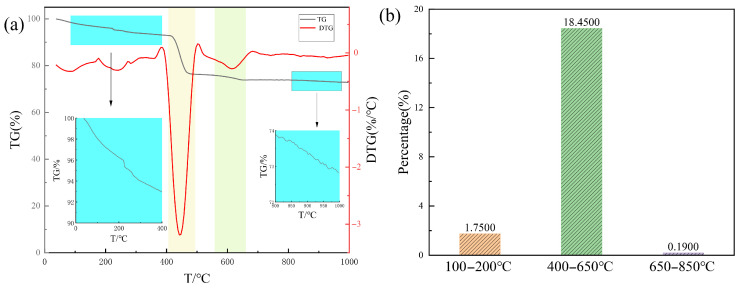
(**a**) TG/DTG curve of healing products; (**b**) mass loss percentage of healing products at different temperatures.

TG was employed to ascertain the composition of diverse healing agents. Figure 17 demonstrates a notable mass reduction occurring within the temperature range of 420–470 °C, which is due to the degradation and carbonation processes of the PANa polymer chain. The weight loss within the scope of 100–200 °C is due to the loss of the bound water of C-S-H gels, as reported in refs. [49,50]. Additionally, the temperature range of 400–650 °C corresponds to the decomposition of CH [51], while the interval of 650–850 °C is associated with the decomposition of CaCO_3_ [52]. By integrating the findings from the TG, FTIR, and XRD analyses, the healing products primarily comprise CH, C-S-H, slight CaCO_3_, and PANa.

## 4. Conclusions

A cement–polymer composite, exhibiting impressive resistance, flexural strength, and self-healing capabilities, was developed by incorporating the in situ polymerization of ANa. A comprehensive investigation was conducted to explore the reinforcing and healing mechanisms. The following conclusions can be drawn:

(1) The polymerization of ANa in the pore solution was confirmed. FTIR spectra show the disappearance of the C=C bond characteristic peak in ANa, while Raman spectra indicate a decrease in the intensity of C=C bond peaks and an increase in C-C bond peaks after polymerization. These results confirm the successful formation of PANa in the pore solution. Additionally, SEM observations of the porous network structure of PANa confirm its binding ability to calcium ions.
(2) The in situ polymerization of the polymer network achieves high-efficiency toughening and late strengthening of the cement. The flexural strength of CSPA-3 surpassed the blank cement sample, achieving 204% of the blank cement sample’s flexural strength after curing for merely 3 days.
(3) The bridge between the polymer network and cement substrate reinforced their bonding performance and made the hydration products form a more dense structure. In addition, the decreased aperture and graded hydration system of CSPA-3 promoted the development of the compressive strength.

Apart from exhibiting high efficiency in reinforcement and toughening, polymer networks polymerized in situ can facilitate the self-healing of cement cracks. The cracks on the CSPA-3 sample almost healed after 28 days of curing with a higher repairing efficiency than blank cement. The healing products contained C-H, C-S-H, CaCO_3_, and ettringite.

## Figures and Tables

**Table 1:** Summary and analysis of properties of polymer cement-based composites in recent years.

Methods	Compressive Strength (28 d)	Flexural Strength (28 d)	Self-Healing Efficiency	Ref.
Through an innovative biomimetic layered structure design, a multi-layered cement-hydrogel composite material was constructed.	/	Increased by 107%	/	[18]
A biomimetic anisotropic hydrogel, namely a alginate/polyacrylamide/halloysite nanotube hybrid hydrogel (SA/AM/HNTs-RDC), was prepared as a self-healing agent.	Decreased by 42%	/	The recovery rate of compressive strength was approximately 92.8%.	[19]
A plug-in network structure was developed through the in situ polymerization of zirconium phosphate and acrylamide.	Decreased	Increased by 105%	/	[20]
In the ordinary Portland cement system, an organic network of the cementitious matrix was prepared through the in situ polymerization of sodium acrylate monomers.	Increased by 15%	Increased by 200%	/	[22]
The in situ polymerization-modified cement paste with different amounts of ANa monomers was used to regulate the rheological behavior.	/	/	/	[24]
By using sodium silicate, sodium silicate/sodium acrylate (ANa), and sodium silicate/sodium polyacrylate as healing agents, efficient healing of existing cracks was achieved through the synergistic reaction between the activation of the cement matrix and the organic polymerization of sodium acrylate.	/	/	The recovery rate of compressive strength was approximately 119.99%	[26]

**Table 2:** Chemical composition of G-class oil well cement.

Components	CaO	SiO_2_	Fe_2_O_3_	SO_3_	Others
Content/%	61.39	21.36	4.56	3.96	4.77

**Table 3:** Mix design of CSPA.

	Cement/(g)	M/(g)	I/(mg)	I′/(mg)	Water/(g)
CSPA-1 (Blank)	800	0	0	0	352
CSPA-2 (2%M)	800	16	288	240	352
CSPA-3 (4%M)	800	32	576	480	352
CSPA-4 (8%M)	800	64	1152	960	352

**Table 4:** Cement samples for tests.

Test	Mold Size/mm^3^	Total Curing Time/d
Flexural strength	40 × 40 × 100	3
Compressive strength	50.8 × 50.8 × 50.8	28
TG/BET (hydration products)	50.8 × 50.8 × 50.8	28
SEM (hydration products)	50.8 × 50.8 × 50.8	28
Self-healing efficiency	18 × 16 × 18	28
SEM/TG/XRD/FTIR (healing product)	18 × 16 × 18	28

**Table 5:** Fitting calculation result of Raman spectra.

0 h			1.5 h			3 h		
Peak Weighting Center/(cm^−1^)	Peak Area Percentage/(%)	Standard Error	Peak Weighting Center/(cm^−1^)	Peak Area Percentage/(%)	Standard Error	Peak Weighting Center/(cm^−1^)	Peak Area Percentage/(%)	Standard Error
1642.31	31.67	0.06028	1642.90	15.27	0.03512	1642.69	23.13	0.07638
1556.75	1.46	0.03000	1559.36	3.20	0.09165	1560.06	3.67	0.08145
1432.60	29.63	0.03000	1432.46	21.71	0.02082	1432.97	19.81	0.05508
1365.43	5.17	0.08622	1364.86	3.12	0.04000	1356.41	0.69	0.02082
1283.27	21.18	0.03215	1284.72	12.18	0.04041	1283.17	13.68	0.04041
1067.42	3.45	0.01528	1069.85	4.23	0.06110	1071.87	6.15	0.03786
900.39	7.44	0.04509	872.00	40.29	0.05508	876.33	32.88	0.12858

**Table 6:** The pore data of CSPA-1 and CSPA-3.

Sample	Curing Time/d	Surface Area (m^2^/g)	Total Pore Volume of Pores (cm^3^/g)	Average Pore Diameter (nm)
CSPA-1	3	15.4411	0.07953	20.6022
	14	28.1092	0.08849	12.5924
	28	31.0427	0.08939	11.5183
CSPA-3	3	5.5207	0.04133	29.9455
	14	9.2676	0.05787	24.9773
	28	19.7404	0.1221	24.7411

**Table 7:** The percentage of mass loss of CSPA-1 and CSPA-3 in different weight loss stages.

Sample	Curing Time/d	Percentage of Mass Loss/%		
		100–200 °C	400–440 °C	550–650 °C
CSPA-1	3	3.25	3.37	1.15
	14	3.63	3.61	1.46
	28	3.64	4.67	1.51
CSPA-3	3	1.12	0.3	2.38
	14	1.31	0.31	2.50
	28	3.28	3.55	3.25

## Data Availability

The original contributions presented in this study are included in the article. Further inquiries can be directed to the corresponding author.
